# The impact of attack frequency and duration on neurocognitive processing in migraine sufferers: evidence from event-related potentials using a modified oddball paradigm

**DOI:** 10.1186/s12883-019-1305-7

**Published:** 2019-04-27

**Authors:** Yunliang Guo, Qiang Tian, Song Xu, Mimi Han, Yue Sun, Yan Hong, Xunyao Hou, Xueping Liu

**Affiliations:** 1 Department of Senile Neurology, Shandong Provincial Hospital Affiliated to Shandong University, Jinan, 250021 Shandong People’s Republic of China; 2 Anti-Aging Monitoring Laboratory, Shandong Provincial Hospital Affiliated to Shandong University, Jinan, 250021 Shandong People’s Republic of China; 3 Department of Anti-Aging, Shandong Provincial Hospital Affiliated to Shandong University, Jinan, 250021 Shandong People’s Republic of China; 4 Department of Geriatrics, Taian City Central Hospital, Taian, 271000 Shandong People’s Republic of China; 5 Department of Neurology, Affiliated Hospital of Shandong Academy of Medical Science, Jinan, 250031 Shandong People’s Republic of China

**Keywords:** Migraine, Event-related potential, P3, Impaired visual spatial attention, Attack frequency, Attack duration

## Abstract

**Background:**

Several studies have suggested that migraineurs suffer from neurocognitive abnormalities, but this phenomenon and exact mechanisms remain controversial. In this study, we aimed to reevaluate visual spatial attention via event-related potential (ERP) examinations and explore further correlations between ERP data and migraine characteristics.

**Methods:**

Altogether, 25 migraine patients (9 males, 16 females; mean age 35.240 years) in the interictal period and 21 age-matched healthy controls (8 males, 13 females; mean age 35.286 years) were recruited. A modified visual oddball paradigm which contained standard, target and novel stimuli was used in the test, and amplitudes and latencies of corresponding original/difference ERP components were measured and analyzed independently.

**Results:**

We found that P3 amplitude was markedly reduced in migraineurs. This phenomenon was further validated in analysis of difference P3 components (target minus standard and novel minus standard). Additionally, the N1 and N2 latencies elicited by novel stimulus were both delayed in patients compared with controls. Furthermore, these deviant cognitive ERPs were correlated with frequency and duration of migraine attacks.

**Conclusions:**

These results indicated impaired visual spatial attention in migraine patients, which could be related to frequency and duration of attacks.

## Background

Migraine is one of the most common and debilitating pain disorders affecting up to 15% of the population [1], mainly causing social functioning impairment, lost productivity and a lower overall quality of life [2]. Migraine clinically manifests with recurrent attacks of headache accompanied by gastrointestinal and autonomic nervous system symptoms, such as nausea and photophobia [3], while the exact pathogenesis has yet to be clearly elucidated.

Some investigations suggest that migraine sufferers could have cognitive impairment, including attentional deficits, visual-spatial processing abnormalities and memory disturbances [4–6]. A nationwide retrospective cohort study has shown that migraine is associated with a higher risk of dementia [7], and an increased prevalence of stroke is found in migraineurs [8], which may lead to neurocognitive abnormalities in succession [9]. Migraine is also closely related to an elevated risk for gray matter volume reduction in frontal cortex and cingulate gyrus, deep white matter hyperintensities, and subclinical brain lesions [10–12], which can all result in cognitive decline [13]. However, several researches demonstrate no association existing between migraine and cognitive function [14, 15]. Hence, it is necessary to fully illustrate the relationship between migraine and cognitive processing, as well as underlying mechanisms.

Event-related potential (ERP), a kind of special evoked potential, can exhibit stimulus-related postsynaptic activity in cerebral regions and reflect corresponding neuroelectrophysiological alterations during cognitive processing. On account of objectivity, noninvasiveness and ultrahigh temporal resolution, ERP is ideally appropriate for investigating specific function of cerebral regions involved in a cognitive task, and has been applied in various neurological disorders, including migraine [16, 17]. Moreover, P3 (also called P300) is undoubtedly the most investigated wave for assessing cerebral information processing, due to its wide scalp distribution, simple recording and high reliability [18, 19].

As conflicting results emerge and underlying mechanisms remain unclear, it is in urgent need to further investigate neurocognitive processing in migraine sufferers. In the present work, we aimed to observe specific patterns of ERP waveforms and components elicited by a modified visual oddball paradigm in migraineurs, and explore correlations between attentional ERP components and clinical characteristics. We hypothesized that migraine patients suffered from attentive processing impairment and corresponding ERP abnormalities.

## Methods

### Subjects and criteria

In this study, 25 migraine patients (9 males, 16 females; mean age 35.240 years) were recruited from Shandong Provincial Hospital Affiliated to Shandong University, 7 with aura and 18 without aura. In addition, 21 age-matched healthy controls (8 males, 13 females; mean age 35.286 years) without headache also participated in the research, which were recruited from hospital staff or community.

Migraineurs were clinically diagnosed according to the beta version of the International Classification of Headache Disorders, 3rd edition (ICHD-3 beta), and were outside migraine attacks during experimental procedures. All patients received no prophylactic anti-migraine therapy, and were drug-free for at least 72 h prior to experiment. Patients with a history of analgesic drug overuse or addiction, mixed headache types, other neurological disorders, such as stroke and brain injuries, were excluded. We also excluded patients with abnormal findings on neurological examinations or brain morphology indicating other potential neurological diseases. Furthermore, all participants were verified to be literate, and had normal or corrected-to-normal vision. Drug/substance abuse, suicide ideation and/or previous suicide attempts were exclusion criteria as well. Migraine characteristics were obtained by interview, including migraine history, frequency and duration of attacks in the previous year, and headache score which represented a scoring of the most severe migraine experienced over the past year by visual analog scale (VAS), with 0 representing no pain and 10 worst possible pain respectively.

We initially enrolled 33 migraine patients (19 females) and 23 age-matched healthy controls (14 females) in the study. Nevertheless, 8 patients (3 females) had to be excluded-two for excessive artefacts (blink and electromyographic activity) within electroencephalogram data and six for incomplete or ambiguous clinical characteristics. So only 25 migraineurs (16 females) were included. In terms of controls, 2 participants (1 female) were excluded due to technical problem during recording. Thus, we finally included 21 healthy controls (13 females) for further analysis.

### Evaluation of emotional state

Emotional state of participants was evaluated by Self-Rating Anxiety Scale (SAS) and Self-Rating Depression Scale (SDS) as suggested in our previous study [20]. Score of SAS above 49 and score of SDS above 52 were considered as anxious and depressed, respectively [21].

### Stimuli and procedure

The experiment was performed in an electrical shielded and quiet room with dim light. The subjects were seated in a comfortable armchair and were instructed to fix their eyes on a fixation cross at the center of a computer monitor (23 in.) placed 50 cm in front of them. A practice run was carried out to ensure that all participants understood this task, and 3 separate blocks of 501 stimuli in total were presented in random order. A modified visual oddball paradigm was used in this experiment, which consisted of standard (smaller circle, *p* = 0.76, *n* = 381), target (larger circle, *p* = 0.12, *n* = 60) and novel (square, *p* = 0.12, *n* = 60) stimuli. The duration of each stimulus was 400 ms, and the inter-stimulus interval was fixed at 400 ms. All subjects were not informed about the incidence of different stimuli, and were directed to discriminate larger circles (target stimuli, *n* = 60, 20 for each block) as accurately and quickly as possible when the paradigm was presented. Participants were required to remember the amount of larger circles they viewed in every block by mental counting and report to experimenter, so that accuracy could be calculated and recorded. Accuracy was defined as the difference between reported number and exact number, and subjects with accuracy below 90% were removed from analysis.

### Electroencephalogram recordings

The electroencephalogram (EEG) of all participants was continuously recorded using Ag-AgCl active electrodes placed in accordance with the 10–20 international system via Neurolab EEG/ERPs 32 Channel Amplifier. Recordings were obtained from midline Fz, Cz and Pz electrode sites, each referenced to left mastoid signals (right mastoid as ordinary recording site). The electrode sites on the scalp were prepared with alcohol and scraped with scrub cream to remove cutin, thus electrode impedance could be maintained below 10 kΩ throughout the experiment. In addition, electrodes placed supraorbitally to the right eye and 10 mm from the right outer canthus were used to record eye movements and remove electrooculogram (EOG) artifacts subsequently. The sampling rate was 1000 Hz, and the low pass filter was set at 100 Hz.

### EEG data preprocessing and measurements

We used ASA 4.9.3 software to analyze EEG data off-line. The data were re-referenced to the average of left and right mastoid signals. EOG artifacts were corrected using the method proposed by Jung et al. [22], and the bandpass filter was set at 0.1–30 Hz (24 dB/octave) for data preprocessing. Then epochs were extracted from 200 ms pre-stimulus to 1000 ms post-stimulus. A − 200 to 0 ms pre-stimulus baseline was applied for all ERP waveform corrections and measurements. Trials with signal exceeding ±100 μV amplitude in any recording channel were rejected from averaging. Finally, segments of standard, target and novel stimuli were averaged separately, and single-subject waveforms were used to generate group-averaged waveforms for further analysis.

**Fig. 1:**
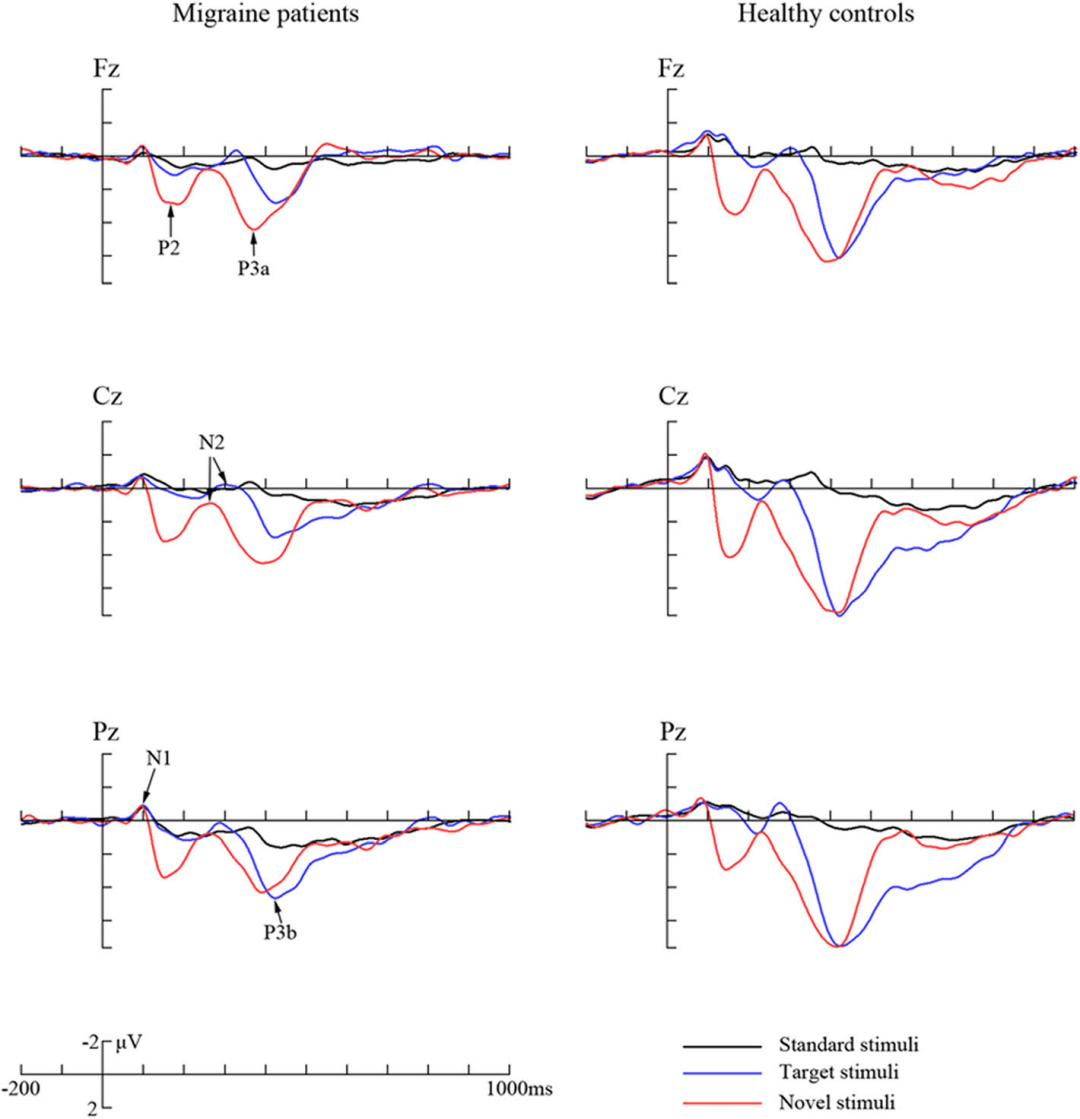
The grand averaged ERP waveforms in migraine patients and healthy controls, respectively. The sites include scalp midline electrodes Fz, Cz and Pz. Original waveforms are elicited by standard (black), target (blue) and novel stimuli (red line)

The peak amplitudes and latencies of ERP components were measured relative to the pre-stimulus baseline period. The positive peak that appeared between 300 and 500 ms post-stimulus was used to define the P3 component. Additionally, the negative peak between 50 and 190 ms, the positive peak between 110 and 270 ms, and the negative peak between 210 and 370 ms were used to define the N1, P2 and N2 components, respectively (see Fig. 1).

To reliably observe and better assess the target and novel effects, the difference waveforms were calculated by subtracting ERPs in response to standard stimuli from those in response to target and novel stimuli, respectively, and corresponding components were further analyzed in difference waveforms (Fig. 3).

### The analyses and measurements were performed blindly by the examiners

#### Statistical analysis

Quantitative data were expressed as mean ± standard deviation (SD). The comparisons of two groups in demographic features, emotional characteristics and behavioral performance were analyzed by Student’s *t*-test for independent samples or *χ*^*2*^ test (two-tailed). Repeated-measures analysis of variance (ANOVA) was used to analyze amplitudes and latencies of original ERP components, with stimulus (standard, target and novel) and electrode (Fz, Cz and Pz) as within-subject factors, while with group (migraine patients vs. healthy controls) as a between-subject factor. Notably, P3 elicited by target and novel stimuli was defined as P3b and P3a components, respectively, and the method applied to analyze P3 amplitudes and latencies was same as mentioned above. For difference ERP components, repeated-measures ANOVA was performed with electrode (Fz, Cz and Pz) as a within-subject factor, while with group (migraine patients vs. healthy controls) as a between-subject factor. The degrees of freedom were corrected using Greenhouse–Geisser epsilon in the case of a sphericity assumption violation. Further post-hoc analysis using Bonferroni correction was conducted if necessary. Moreover, Pearson product-moment correlation coefficients (*r*) were calculated and reported to represent correlations between clinical variables and electrophysiological data. All statistical analyses were conducted with SPSS 23.0 (SPSS Inc., Chicago, IL, USA), and results with *p* < 0.05 were considered to be significant. Effect sizes of remarkable results were reported as partial eta squared (*η*^2^).

## Results

### Sample characteristics

The demographic and clinical characteristics of subjects are shown in Table 1. The age, gender and education level were not significantly different between two groups (all *p* > 0.6). In this study, migraineurs experienced 3.160 (SD 2.257) times of attacks per month, with each lasting for 20.320 h (SD 21.205). On average, they had been suffering from migraine for 8.010 years (SD 6.522), and average headache score was 5.360 (SD 1.091). As for emotional characteristics, assessed by SAS and SDS, migraine patients tended to be more anxious and depressed compared with healthy controls (*p* < 0.001 and *p* = 0.001 respectively).

**Table 1:** Sample characteristics

	Migraine patients	Healthy controls	*t*/*χ*^*2*^	*P*
N	25	21		
Age, years	35.240 ± 11.336	35.286 ± 9.233	− 0.014	0.989
Age range, years	20–58	24–55		
Gender, male/female	9/16	8/13	0.022	0.883
Education, years	14.320 ± 3.484	14.952 ± 4.488	−0.526	0.602
Migraine frequency, times per month	3.160 ± 2.257	–	–	–
Duration of migraine, Hours	20.320 ± 21.205	–	–	–
History of migraine, Years	8.010 ± 6.522	–	–	–
Headache score, 0–10	5.360 ± 1.091	–	–	–
SAS score	47.520 ± 9.575	35.476 ± 7.481	4.583	0.000***
SDS score	46.440 ± 10.737	35.857 ± 8.785	3.534	0.001**

Data were expressed as mean ± SD

*SAS* Self-Rating Anxiety Scale, *SDS* Self-Rating Depression Scale

**P < 0.01, ***P < 0.001 by Student’s *t*-test (two-tailed)

### Behavioral performance

There was no remarkable difference in accuracy between two groups (migraineurs, 97.7%; healthy controls, 98.4%; *t* = − 1.244, *p* = 0.220).

### Characterization of original ERP data

The grand averaged ERP waveforms are shown in Fig. 1, and analyses of multiple original ERP components are summarized in Table 2.

**Table 2:** Results and analyses of original ERP components

		Migraine patients			Healthy controls			Statistics	
Original								Group	Group × Stimulus
ERPs		Standard	Target	Novel	Standard	Target	Novel	*F*	*F*
P3	Amplitude (μV)	2.243	4.828^a^	5.665^b^	2.263	8.402^a^	8.416^b^	12.278**	6.261**
		(1.800)	(3.001)	(3.180)	(1.426)	(3.977)	(4.985)		
	Latency (ms)	426.720	431.920^a^	405.693^b^	432.397	430.095^a^	401.302^b^	0.001	0.547
		(39.785)	(31.326)	(39.050)	(37.928)	(30.989)	(37.246)		
N1	Amplitude (μV)	−1.437	−1.623	−2.120	−2.093	−2.587	−2.941	3.922	0.206
		(1.468)	(1.663)	(2.367)	(1.319)	(2.324)	(2.353)		
	Latency (ms)	117.653	115.493	111.027	122.381	117.714	99.032	0.080	4.818*
		(29.340)	(32.314)	(27.180)	(30.310)	(31.819)	(23.011)		
P2	Amplitude (μV)	1.685	1.942	4.564	1.616	2.614	5.766	1.547	1.566
		(1.713)	(1.831)	(3.550)	(1.656)	(2.218)	(3.198)		
	Latency (ms)	213.453	217.560	180.827	205.937	210.111	168.095	2.697	0.177
		(34.593)	(32.282)	(39.135)	(29.831)	(29.958)	(34.486)		
N2	Amplitude (μV)	−0.618	−1.086	−0.362	−1.470	−1.235	−0.207	0.370	1.484
		(1.654)	(2.058)	(2.741)	(2.108)	(2.021)	(2.551)		
	Latency (ms)	303.253	292.440	269.080	310.635	281.349	254.016	1.561	3.407*
		(32.840)	(29.347)	(23.952)	(45.156)	(29.665)	(36.216)		

SD appeared in parenthesis of the mean

^a^Target-elicited P3b; ^b^ novel-elicited P3a

*P < 0.05, **P < 0.01 by repeated-measures ANOVA (Bonferroni correction)

#### P3 component

**Fig. 2:**
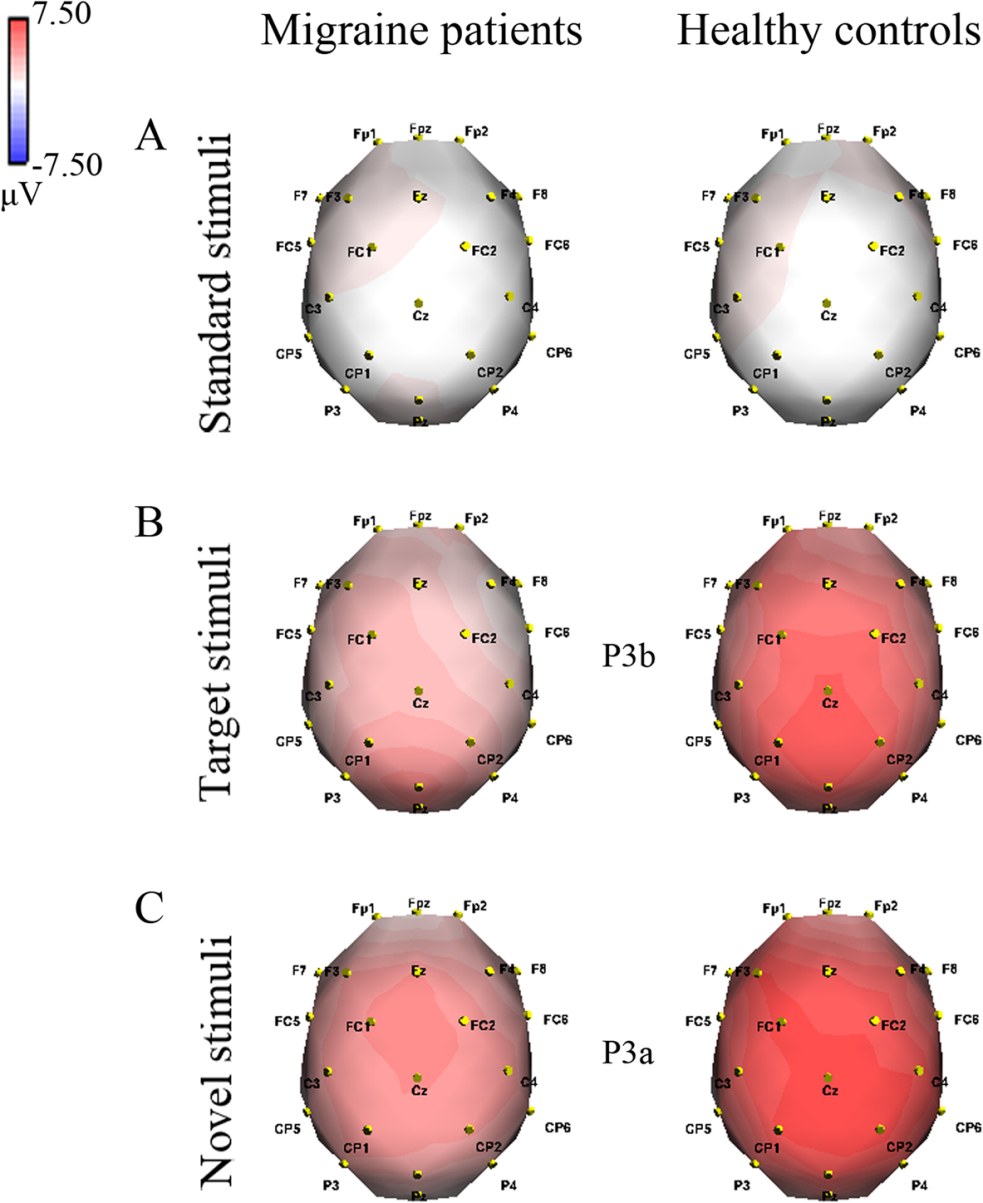
Topographical voltage distributions of P3 component in migraine patients and healthy controls, respectively. P3 is elicited by different stimuli. **a** Standard stimuli at 425–435 ms; **b** target stimuli (P3b) at 430–440 ms; **c** novel stimuli (P3a) at 400–410 ms. Red denotes a positive and blue a negative potential

Figure 2 depicts topographies of voltage distribution for P3 component elicited by standard, target (P3b) and novel stimuli (P3a). Migraineurs displayed lower P3 amplitude (4.245 ± 3.085 μV) than healthy controls (6.360 ± 4.746 μV, *F*(1,44) = 12.278, *p* = 0.001, partial *η*^2^ = 0.218). Across groups, the main effect of stimulus was significant (*F*(1.885,82.944) = 50.827, *p* < 0.001, partial *η*^2^ = 0.536), with a maximum of 7.040 μV for novel-elicited P3a. Furthermore, there was a remarkable group × stimulus interaction (*F*(1.885,82.944) = 6.261, *p* = 0.003, partial *η*^2^ = 0.125) (Table 2), and further post-hoc analysis revealed marked differences in P3b and P3a amplitudes between two groups (*F*(1,44) = 16.057, *p* < 0.001, partial *η*^2^ = 0.267 for P3b; *F*(1,44) = 6.061, *p* = 0.018, partial *η*^2^ = 0.121 for P3a), while not for standard stimulus (*F*(1,44) = 0.003, *p* = 0.958) (see Fig. 2). Additionally, we compared P3b and P3a amplitudes between groups at each electrode, and all results reached significant levels (*p*s < 0.04) except for P3a at Fz (*F*(1,44) = 3.829, *p* = 0.057). The amplitude of P3 showed noticeable main effect of site (*F*(1.578,69.437) = 5.883, *p* = 0.008, partial *η*^2^ = 0.118). Moreover, difference between the location of recording was not observed: left (F3, C3 and P3) vs. right (F4, C4 and P4) vs. midline (Fz, Cz and Pz) (*p*s > 0.05).

In terms of P3 latency, remarkable stimulus effect (*F*(1.852,81.479) = 19.174, *p* < 0.001, partial *η*^2^ = 0.304) was obtained, of which novel-elicited P3a was fastest (403.497 ms). The analysis did not reveal other significant results (*p*s > 0.05).

#### N1 component

We did not find notable effects or interactions in N1 amplitude (*p*s > 0.05).

As shown in Table 2, although there existed no difference between groups in N1 latency (*F*(1,44) = 0.080, *p* = 0.779), the two-way interaction of group × stimulus appeared remarkable (*F*(1.524,67.037) = 4.818, *p* = 0.018, partial *η*^2^ = 0.099), and post-hoc analysis discovered that the latency elicited by novel stimulus was obviously prolonged in migraineurs (*F*(1,44) = 4.709, *p* = 0.035, partial *η*^2^ = 0.097), while this phenomenon was not observed for standard (*F*(1,44) = 0.490, *p* = 0.488) and target stimuli (*F*(1,44) = 0.077, *p* = 0.783).

#### P2 component

Neither group effects (*F*(1,44) = 1.547, *p* = 0.220 for amplitude; *F*(1,44) = 2.697, *p* = 0.108 for latency) nor group × stimulus interactions (*F*(1.186,52.196) = 1.566, *p* = 0.219 for amplitude; *F*(1.593,70.094) = 0.177, *p* = 0.788 for latency) was observed significant in analysis of P2 component. Other effects or interactions did not reach significant levels either (*p*s > 0.05).

#### N2 component

No statistically significant difference was obtained in N2 amplitude (*p*s > 0.05).

As for N2 latency, it did not differ between groups (*F*(1,44) = 1.561, *p* = 0.218), but the noticeable main effect of stimulus (*F*(1.839,80.908) = 9.162, *p* < 0.001, partial *η*^2^ = 0.528) and interaction of group × stimulus (*F*(1.839,80.908) = 3.407, *p* = 0.042, partial *η*^2^ = 0.072) was found. It was noteworthy that only latency elicited by novel stimulus was markedly delayed in patients compared with controls (*F*(1,44) = 4.398, *p* = 0.042, partial *η*^2^ = 0.091).

### Characterization of difference ERP data

**Fig. 3:**
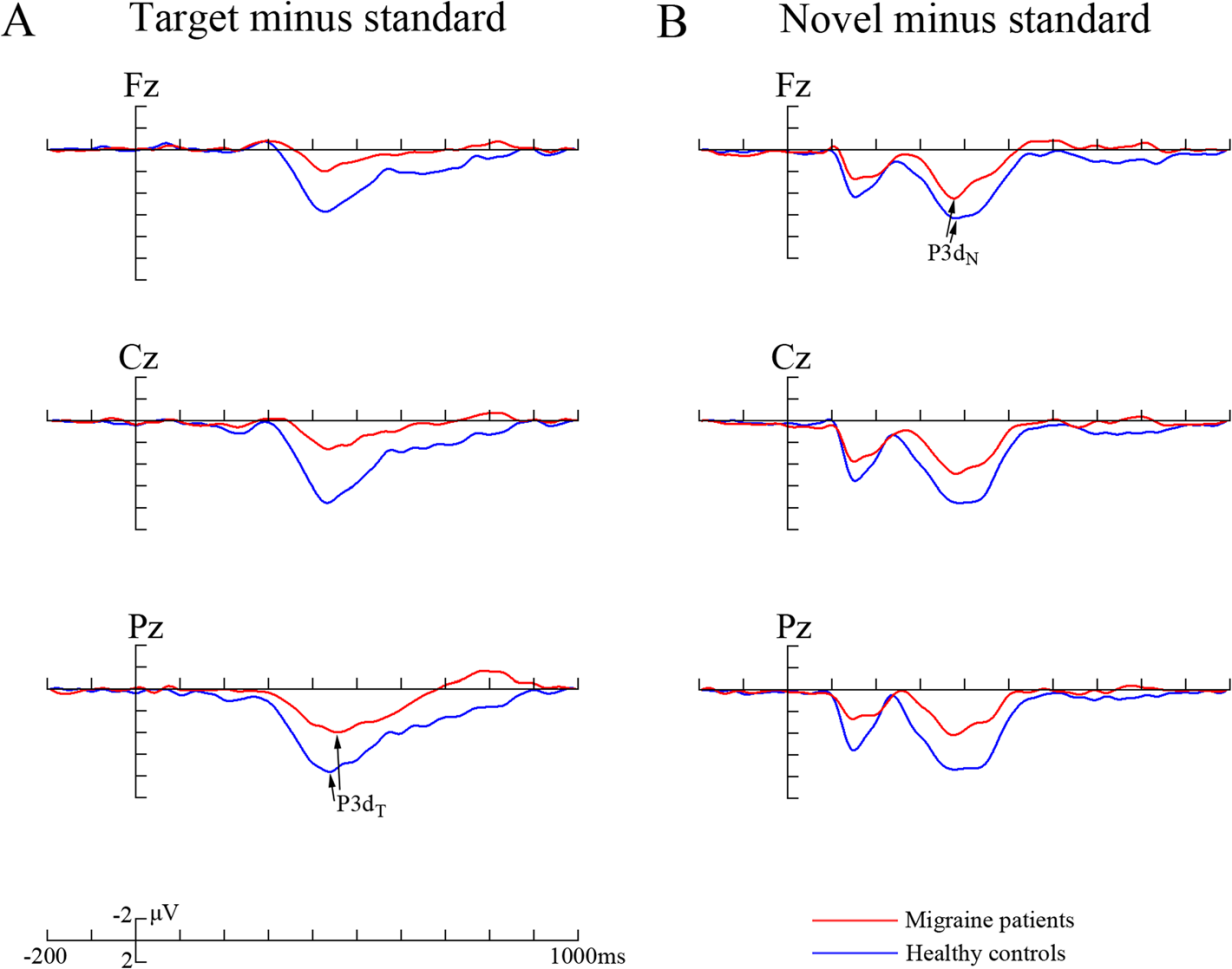
The averaged difference waveforms in migraine patients and healthy controls, respectively. The sites include scalp midline electrodes Fz, Cz and Pz. **a** Target minus standard difference ERPs; **b** novel minus standard difference ERPs. P3d_T_ and P3d_N_ represent the P3 target and novel effects, respectively. Red line represents migraine patients and blue line healthy controls

**Fig. 4:**
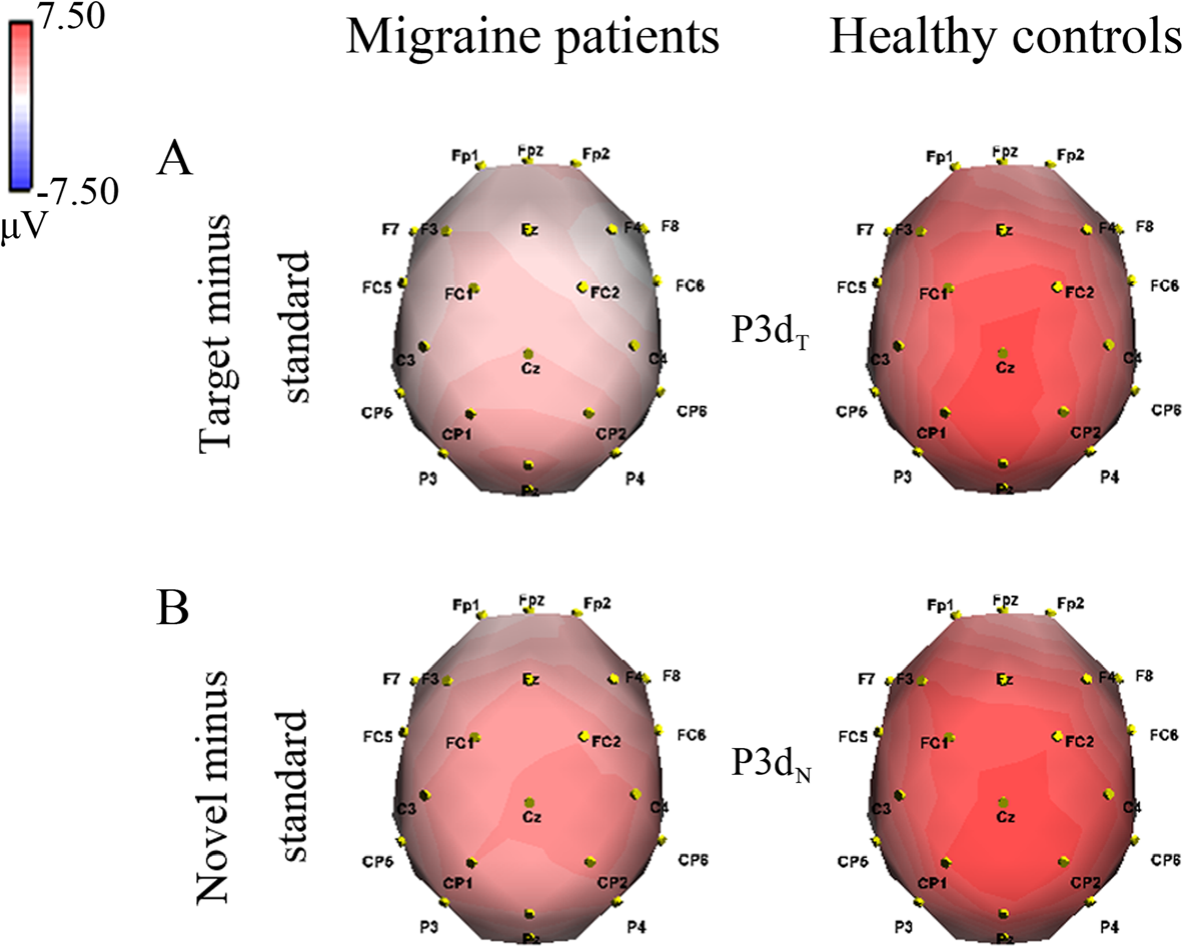
Topographical voltage distributions of difference P3 components in migraine patients and healthy controls, respectively. **a** P3d_T_ at 420–430 ms; **b** P3d_N_ at 380–395 ms. P3d_T_ and P3d_N_ represent the P3 target and novel effects (target minus standard and novel minus standard), respectively. Red color denotes a positive and blue color a negative potential

The difference waveforms are demonstrated in Fig. 3, in which P3 difference components were further analyzed (Table 3 and Fig. 4).

**Table 3:** Results and analyses of difference P3 components

		Migraine patients			Healthy controls			Statistics	
Difference								Group	Group × Electrode
ERPs		Fz	Cz	Pz	Fz	Cz	Pz	*F*	*F*
P3d_T_	Amplitude (μV)	3.198	3.702	5.326	6.689	8.543	8.633	35.905***	3.416*
		(1.505)	(1.594)	(2.373)	(2.515)	(3.315)	(3.473)		
	Latency (ms)	417.560	430.440	434.720	422.095	438.333	423.286	0.002	1.429
		(39.528)	(34.141)	(38.806)	(32.695)	(31.806)	(30.798)		
P3d_N_	Amplitude (μV)	5.246	5.440	5.354	7.675	9.202	9.014	20.445***	3.397*
		(2.251)	(2.196)	(2.209)	(2.904)	(3.294)	(3.295)		
	Latency (ms)	377.960	384.520	390.320	387.524	385.429	397.476	0.549	0.531
		(30.748)	(37.047)	(34.329)	(26.506)	(27.298)	(31.295)		

SD appeared in parenthesis of the mean

P3d_T_ and P3d_N_ represent the P3 target and novel effects (target minus standard and novel minus standard), respectively

*P < 0.05, ***P < 0.001by repeated-measures ANOVA (Bonferroni correction)

#### P3d_T_ and P3d_N_ components

We measured and analyzed P3d_T_ (target minus standard) and P3d_N_ (novel minus standard) components to better evaluate the P3 target and novel effects, respectively. The analysis of amplitudes both demonstrated significant electrode effects (*F*(1.845,81.183) = 20.346, *p* < 0.001, partial *η*^2^ = 0.316 for P3d_T_; *F*(1.857,81.705) = 5.283, *p* = 0.008, partial *η*^2^ = 0.107 for P3d_N_). The amplitudes were smaller for migraine patients (P3d_T_: 4.075 ± 1.516 μV, P3d_N_: 5.347 ± 2.015 μV) than for healthy controls (P3d_T_: 7.955 ± 2.787 μV, P3d_N_: 8.631 ± 2.893 μV; *F*(1,44) = 35.905, *p* < 0.001, partial *η*^2^ = 0.449 for P3d_T_; *F*(1,44) = 20.445, *p* < 0.001, partial *η*^2^ = 0.317 for P3d_N_), and the significant interactions of group × electrode (*F*(1.845,81.183) = 3.416, *p* = 0.041, partial *η*^2^ = 0.072 for P3d_T_; *F*(1.857,81.705) = 3.397, *p* = 0.042, partial *η*^2^ = 0.072) were also observed (see Table 3 and Fig. 4). Subsequent comparisons indicated that patients exhibited reduced P3d_T_ and P3d_N_ amplitudes in all electrodes (*p*s < 0.004).

We failed to find significant difference in latencies of P3d_T_ and P3d_N_ (all *p* > 0.05).

### Correlations between attentional ERP components and clinical characteristics

Based on the aforementioned results, P3 amplitude, N1 and N2 latencies elicited by different stimuli, as well as amplitudes of P3d_T_ and P3d_N_, were selected to further investigate the relationships between electrophysiological data and clinical features in migraineurs. It was discovered that P3b and P3d_N_ amplitudes showed significant, moderate and negative correlations with migraine frequency. In addition, N1 latency-novel and N2 latency-target were also positively correlated with migraine frequency. We also observed significant relationships between amplitudes of P3b, P3a and P3d_N_, N1 latency-standard, N2 latency-novel and duration of migraine, whereas the remaining ERP data did not correlate with migraine characteristics (Table 4).

**Table 4:** Correlations between attentional ERP components and clinical characteristics in migraine patients

	Migraine frequency	Duration of migraine	Headache score	Migraine history	SAS score	SDS score
Original/difference ERPs	*r* (*p* value)	*r* (*p* value)	*r* (*p* value)	*r* (*p* value)	*r* (*p* value)	*r* (*p* value)
P3 amplitude-standard	−0.390 (0.054)	0.111 (0.598)	−0.165 (0.432)	− 0.023 (0.914)	−0.061 (0.772)	−0.003 (0.989)
P3 amplitude-target^a^	−0.417 (0.038)*	−0.401 (0.047)*	−0.212 (0.309)	−0.141 (0.502)	0.076 (0.717)	−0.044 (0.835)
P3 amplitude-novel^b^	−0.104 (0.620)	−0.426 (0.034)*	−0.176 (0.400)	0.031 (0.882)	−0.061 (0.771)	−0.195 (0.351)
N1 latency-standard	−0.178 (0.394)	0.400 (0.048)*	0.209 (0.315)	0.112 (0.596)	−0.063 (0.763)	0.047 (0.823)
N1 latency-target	0.044 (0.835)	−0.166 (0.427)	0.005 (0.982)	0.036 (0.864)	0.340 (0.096)	0.410 (0.042)*
N1 latency-novel	0.435 (0.030)*	0.168 (0.423)	0.097 (0.646)	−0.179 (0.392)	0.104 (0.619)	0.057 (0.788)
N2 latency-standard	0.076 (0.720)	0.080 (0.705)	−0.092 (0.663)	0.082 (0.698)	0.090 (0.669)	−0.062 (0.768)
N2 latency-target	0.400 (0.048)*	0.081 (0.699)	0.024 (0.911)	0.140 (0.506)	0.082 (0.695)	0.080 (0.703)
N2 latency-novel	0.001 (0.995)	0.398 (0.049)*	0.329 (0.108)	−0.101 (0.630)	0.087 (0.678)	0.121 (0.566)
P3d_T_ amplitude	−0.056 (0.791)	−0.268 (0.196)	−0.094 (0.656)	−0.137 (0.513)	−0.018 (0.933)	−0.142 (0.497)
P3d_N_ amplitude	−0.405 (0.044)*	−0.440 (0.028)*	−0.281 (0.174)	0.170 (0.415)	−0.019 (0.929)	−0.085 (0.685)

*r* represents Pearson product-moment correlation coefficient

*SAS* Self-Rating Anxiety Scale, *SDS* Self-Rating Depression Scale

^a^P3b amplitude; ^b^ P3a amplitude

P3d_T_ and P3d_N_ represent the P3 target and novel effects (target minus standard and novel minus standard), respectively

*P < 0.05 by Pearson’s correlations (two-tailed)

## Discussion

Migraine is the most common disabling primary headache disorder, not only for a wide range of clinical symptoms related to migraine attacks, but also for its potential cognitive disturbances [4–6, 23]. In this study, by using a three-stimulus visual oddball paradigm, reduced amplitudes of P3, P3d_T_ and P3d_N_ components, together with delayed N1 and N2 latencies elicited by novel stimulus were found in migraineurs. Moreover, these cognitive ERP abnormalities were directly correlated with frequency and duration of migraine attacks. The aforementioned results suggested that migraine patients might suffer from abnormalities in visual spatial attention, such as target processing, orienting responses and speed of visual information processing, especially when triggered by infrequent stimuli, which could be potentially modulated by some clinical characteristics.

Various relatively late and positive components are defined as P3, which could be subdivided as P3b and P3a. This component is an objective index to describe characteristics of cognitive processing, with its amplitude and latency representing intensity and timing of processing respectively [18, 19]. In our study, significant group effect and group × stimulus interaction were found in P3 amplitude, and post-hoc analysis revealed that the decreased amplitude mainly existed in target-elicited P3b and novel-elicited P3a, while the latency did not differ between groups. These findings were further validated in P3d_T_ and P3d_N_ components. Consistently, other researchers observed reduced P3 amplitude in migraine patients, without any alterations in latency [24, 25]. Nevertheless, previous study showed prolonged P3 latency rather than change of amplitude in migraineur group [26]. The discrepancies might arise from various methodologies used for sample selection and migraine assessment, as well as different experimental procedures. Since the amplitude of P3 mainly depends on stimulus relevance, attention allocation, memory and complexity of task [27], our findings indicated that migraineurs might have interictal impairment in visual spatial attention, such as target processing and orienting responses [28].

N1 component depicts the early sensory processing and is usually considered to reflect selective attention which represents the capability of responding to a specific stimulus [29]. N2 component, a negativity with predominant frontal-central distributions, reflects the early target selection, response preparation and conflict detection [30, 31]. Similar results were obtained from analysis of N1 and N2. We found no significant effects or interactions in N1 and N2 amplitudes. In terms of N1 and N2 latencies, the group × stimulus interactions were statistically significant, and were both markedly delayed under novel stimulus in migraine sufferers. The aforementioned results implied that migraineurs might suffer from deficits in the speed of visual information processing, especially when triggered by infrequent or unfamiliar stimulus.

P2 is predominantly distributed in frontal regions with a latency of around 200 ms after stimulus onset, and is believed to index automatic evaluation, task-related classification and attentional recruitment that form bases for subsequent cognitive processing [32, 33]. The frontal area plays a vital role during these processes, and previous researches have demonstrated frontal dysfunction in migraineurs, such as reduced frontal P3a [34] and decreased gray matter density in the frontal lobe [35]. However, out of our expectations, we failed to discover any P2 abnormality in migraine patients. The inconsistencies could be due to different participant inclusion criteria, like broader range of ages in this experiment, and diverse evaluation protocols.

In order to illuminate relationships between attentional ERP components and clinical variables of migraine sufferers, Pearson’s correlations were calculated. Almost no correlations were found between cognitive ERPs and emotional abnormalities. Meanwhile, we discovered that there were negative correlations between P3/difference P3 amplitudes and frequency and duration of attacks, and latencies of N1 and N2 were positively correlated with these characteristics. Likewise, it was verified that the poorer cognitive performance of migraineurs was related to duration and frequency of headache [26], and Calandre et al. also reported that the frequency of migraine attacks could affect visual attention [36]. The existing correlations between ERP data and migraine characteristics might arise from the fact that the experience of headache itself could modulate cognitive performance [37, 38]. Therefore, repeated migraine attacks with longer duration might result in more serious cerebral dysfunction and more severe deficits in neurocognitive processing, at least under attentional conditions.

The standardized subject selection criteria and collection of detailed migraine characteristics were included in the strengths of present study. A three-stimulus oddball paradigm with high sensitivity was used in migraineurs for the first time to investigate visual processing, and we employed difference waveforms together with voltage topographical maps to make the results more convincing. Additionally, to our knowledge, this was also the first study to systemically discuss the correlations between attentional ERP components with elaborate classification and migraine characteristics. Since it has been demonstrated that even subclinical levels of anxiety and depression may impact cognitive processing [39, 40], the influence of emotion on electrophysiological data was excluded in this study. Nevertheless, there were several limitations constraining the interpretation of our findings. Firstly, migraine characteristics, including patients’ sufferings in the previous year, were obtained by interview, which might be affected by recall bias. In this study, we used a structured questionnaire to ask patients and their family members (usually spouses) who were familiar with their complaints simultaneously, and patients whose clinical characteristics were incomplete or ambiguous were excluded in order to diminish the impact of recall bias to the minimum extent. In addition, the smaller P3 amplitudes in migraine patients could also be due to a higher baseline degree of activation. However, compared with healthy controls, reduced amplitudes were only observed for target (P3b) and novel stimuli (P3a) in migraineurs, while not for standard stimuli. These findings were further validated in analysis of subtraction-derived P3d_T_ (target minus standard) and P3d_N_ (novel minus standard) components. Therefore, it appeared that the smaller P3 amplitudes were more likely due to the impaired visual spatial attention in migraineurs rather than the higher activity for baseline. This issue needs further investigation in the future. Furthermore, the small sample size confined the investigation of the effects of age and gender, and the distinctions between migraine with and without aura were not explored either. Finally, we did not perform source analysis of deviant ERP components in migraineurs, which we would work on in the near future to uncover the underlying network-level pathology.

## Conclusions

In this study, we elucidated that migraineurs had reduced P3/P3d_T_ (target minus standard)/P3d_N_ (novel minus standard) amplitudes and prolonged latencies of N1 and N2 under novel stimulus, suggesting the existence of impairment in visual spatial attention, including target processing, orienting responses and speed of visual information processing, especially in response to infrequent stimuli. These cognitive parameters were correlated with frequency and duration of attacks. Given this, in order to facilitate better outcomes for migraine patients, it is important to take neurocognitive processing abnormalities into account, and ERP examinations could be a sensitive method for early diagnosis.

## Abbreviations

ANOVA: Analysis of variance
EEG: Electroencephalogram
EOG: Electrooculogram
ERP: Event-related potential
ICHD-3 beta: Beta version of the International Classification of Headache Disorders, 3rd edition
P3d_N_: P3 novel effect (novel minus standard)
P3d_T_: P3 target effect (target minus standard)
*r*: Pearson product-moment correlation coefficients
SAS: Self-Rating Anxiety Scale
SD: Standard Deviation
SDS: Self-Rating Depression Scale
VAS: Visual analog scale
*η*^2^: Partial eta squared
